# Palmitic acid-modified GnRH-Th epitope peptide immunocastration vaccine (W/O/W adjuvant) can effectively ensure the castration and reduce the smelly smell in boars

**DOI:** 10.3389/fvets.2023.1174770

**Published:** 2023-04-24

**Authors:** Huayi Gao, Kun Liu, Lu Zhang, Yongfang Wang, Xubin Fu, Yujie Guo, Mei Bai, Yanbing Shen, Min Wang

**Affiliations:** ^1^Key Laboratory of Industrial Fermentation Microbiology, Ministry of Education, Tianjin Key Lab of Industrial Microbiology, College of Biotechnology, Tianjin University of Science and Technology, Tianjin, China; ^2^Tianjin Ringpu Bio-technology Co., Ltd., Tianjin, China; ^3^Dr. Bing Zhang Department of Statistics, College of Arts and Sciences, University of Kentucky, Lexington, KY, United States; ^4^Tianjin Customs Animal and Plant and Foodstuffs Inspection Center, Tianjin, China; ^5^Tianjin Agricultural University, Tianjin, China

**Keywords:** immune castration vaccine, gonadotropin release hormone, reduce the smelly smell, gonadotropin-releasing hormone, boar castration

## Abstract

**Introduction:**

Recent studies have demonstrated the effectiveness of Gonadotropin-releasing hormone (GnRH) in inhibiting testicular growth and development in male animals to achieve castration while improving the meat quality of various livestock species, including cattle, sheep, goats, and pigs.

**Methods:**

In this research, a GnRH-Th vaccine was synthesized using the Fmoc solid-phase synthesis technique, and the T helper (Th) antigen was modified with palmitic acid to improve its efficacy. The vaccine was then coated with a water-in-oil-in-water adjuvant to improve stability and safety. After passing safety and stability tests, the vaccine was administered to 13-week-old boars.

**Results:**

The results showed that it was stable, safe, and effective for up to 15 months. Moreover, the vaccine did not negatively affect the growth rate and body weight of the pigs. The palmitic acid-modified “GnRH-Th epitope peptide immunocastration vaccine (Water-in-Oil-in-Water (W/O/W)) effectively reduced the testosterone concentration and achieved castration. The concentration of androstenone and skatole hormones significantly decreased, leading to improved meat quality in the boars. The boars were then slaughtered at 33 weeks of age, and the results showed that the meat quality of the vaccinated boars was superior to that of the non-vaccinated control group (*p* < 0.05).

**Discussion:**

This study demonstrated that GnRH can safely and effectively achieve immune castration in boars after coupling T cell epitopes, palmitic acid modification and W-O-W coating. Provide a better method for the further development of GnRH and the realization of animal welfare.

## 1. Introduction

Castration is widely used in pig production to control odor, but the pain and stress caused by testicular removal are contrary to animal welfare. On January 1, 2018, the European Union banned castration operations on boars (1, 2). In the future, painless immunization is expected to replace surgical castration of animals. Currently, over 60 countries have approved the use of immunization to castrate boars. China, as the world’s largest pig breeder and consumer, produces more than 450 million fattening pigs annually, half of which are castrated boars. Boars require castration during the pig breeding process, as male hormones will accumulate in their bodies after growing to a certain age, causing odor and negatively affecting the flavor and taste of pork. The gonadotropin-releasing hormone GnRH plays a crucial role in animal growth and reproduction by stimulating the pituitary synthesis and secretion of gonadotrophic hormone in the head (3). Multiple factors influence animal reproduction both *in vivo* and *in vitro*, including positive and negative feedback, where the hypothalamic–pituitary-gonadal axis plays an important role, while GnRH is the most critical (4, 5). Injecting the maximum dose of GnRH or its analogs into the animal body significantly decreases the secretion of Follicle-stimulating hormone and Luteinizing hormone, inhibiting the animal’s reproductive system (6). Schanbacher BD found that by injecting GnRH into a horse’s body, the testosterone and LH concentrations remained at low levels, and the GnRH antibody titer increased significantly. About 7 months later, these indicators returned to normal, but after secondary immunization, the GnRH antibody titer quickly recovered, and testosterone concentration dropped to castration level. Thus, immunization against GnRH not only controls animal reproduction but also avoids potential infections and irreversible damage to the reproductive system due to surgical castration.

However, the current immune castration vaccine also has some issues, such as excessive inflammatory response, poor efficacy, and a short duration of the vaccine’s effect. For example, the use of immune castration vaccines may lead to an inflammatory response in the tissue. Molenaar et al. (7) found that after GnRH treatment, tissue sections showed pathological changes, such as cell enlargement, enlarged space, and infiltrating fibroblasts. However, some scholars have observed the reversible recovery of the animal body after immunization (8). The immune castration vaccine only temporarily suppresses the development of the gonadal axis. When the GnRH antibody titer is lower than a certain level, the animal body can recover. In addition, the GnRH molecule is a short-chain peptide with only 10 amino acids, a simple structure, and a small molecular weight (9). It cannot stimulate the humoral immune reaction of the animal body alone to exert the effect of immune castration. Instead, a coupling carrier or structural modification must be used to produce a strong immune response. It is worth noting that the shelf life of the castration vaccine used for odor control in boars needs to be maintained until the animals are fully grown. The typical lifespan of a boar is 6 to 7 months, and the optimal period for immunization with the vaccine is between 8 to 13 weeks of age. The vaccine’s effect should last for at least 8 weeks after the second immunization (10).

In order to address the challenges with the current GnRH immunocastration vaccine, researchers have developed a modified approach. They combined GnRH with the T cell epitope (TCE) on the surface of T cells to create the “GnRH-Th epitope peptide.” This approach stimulates the humoral immunity of the body and results in the production of GnRH antibody (11, 12). Additionally, researchers have screened commercial adjuvants and conducted palmitate modification to extend the vaccine duration and improve the vaccine titer. While the water-in-oil adjuvant used in the GnRH immune potential vaccine has demonstrated significant effectiveness, it has also resulted in side effects such as local swelling, fever, and granuloma formation, with some cases leading to serious ulcers, necrosis, and even death. These issues not only reduce production efficiency but also affect the quality of pork (13). To address these issues, the researchers in this study utilized a water-in-oil-in-water vaccine adjuvant, which causes less stress on the animal body and is less likely to cause bulges and swelling, while also reducing the use of mineral oil. The study found that the GnRH IgG vaccine, combined with the Th epitope peptide and palmitic acid modification, resulted in a 100% castration standard with less severe immune stress on the animals.

## 2. Materials and methods

### 2.1. Primary reagents

Pig HRP-IgG (H + L), By Jinan Baditai Biological Co., LTD.; Porcine growth hormone (GH) ELISA kit, Produced by Nanjing SenBeiJia Biological Technology Co., Ltd.; Oil-in-water adjuvant (MONTANIDEISA61VG), oil-in-water adjuvant (MONTANIDEISA201VG), Produced by French, France; Sheep anti-rat HRP-IgG (H + L), Produced by KPL Company; Testosterone-free kit, Produced by Beijing North Biotechnology Co., Ltd.

### 2.2. Instruments and equipment

PSI (peptide synthesizer model-200) is from Tianjin Sierra Peptide Technology Co., LTD.; High-performance liquid chromatograph (Agilent 1,100) was provided by Agilent Company; Triple four-stage rod tandem mass spectrometer (API 4000) is provided by AB Company; Small high-speed centrifuge (type 5,418), pipette provided by Thermo company; 96-well flat bottom ELISA reaction plate (3590) provided by Corning/Costar; Biochemical incubator (SPX-250) is provided by Ningbo Jiangnan Instrument Factory; Enzyme-linked immunodetector (Infinite 200PRO) is provided by Bio-RAD; Automatic microplate reader (ELX808) is provided by Biotek Company.

### 2.3. Synthesis of GnRH polypeptides and analogs

**Figure 1:**
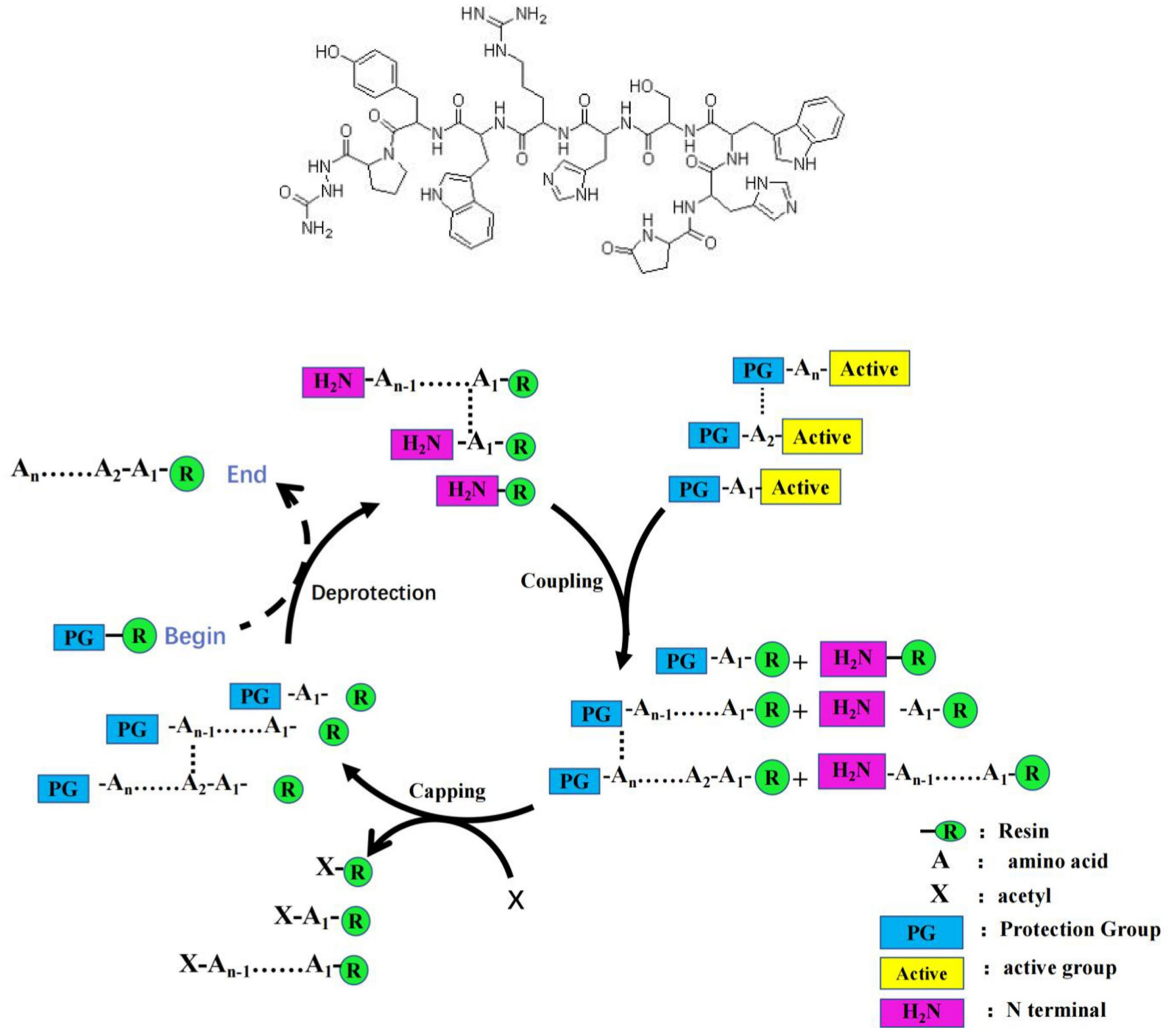
The structure of GnRH (upper) and its synthesis method (lower).

GnRH was synthesized following the given method: 0.8 g MBHA resin was weighted and added into PSI peptide synthesizer, and then swelled by 24 ml of NMP for 2 h. After discarding the residuary NMP, swelled resin was washed by 24 ml fresh NMP for 90 s, and then discarded the washing liquid. Resin was washed for three time, and then treated by 24 mL 20% PIP/NMP for 5 min, then washed by 24 mL NMP for 90 s. Then the resin was treated by 20% PIP/NMP (24 mL) for 10 min. HOBt/NMP solution (1 mol/L) and DIC/NMP (1 mol/L) were placed at room temperature and stirred until dissolved at 10–25°C for 5 min, and then the solution was added into the synthesizer. The first amino acid of GnRH was dissolved in NMP/CH_2_Cl_2_/Triton X-100, and then added into the synthesizer and reacted for 2 h at 25 ~ 35°C. After analyzed by Kaiser method, the reaction product was treated by 24 mL PIP/NMP (20%) for 5 min, discarded the reaction liquid, and then washed by NMP (24 mL) for 90 s. The second amino acid of GnRH was combined on the resin following the same method, then the third one. After all amino acids of GnRH were combined in turn, MeOH (24 mL) was added into the reactor and the product was washed by MeOH for four times, 90 s each time. Resin with peptide GnRH was treated with cutting fluids for 2 h at −20°C, the mixture was filtered and concentrated in vacro by rotary evaporimeter, and then treated by equal petroleum ether to give the peptide. The peptide was collected and washed to discard the impurities, and then lyophilized to give the peptide (see Figure. 1).

Synthesis method of G1: Using Fmoc solid phase synthesis technology to synthesize GnRH polypeptides, the peptide chain of GnRH was extended from C to N using the above established polypeptide synthesis method, using HOBT/DIC/NMP as a coupling agent and using Fmoc SPPS method for synthesis. The linear amino acid sequence of the broad spectrum Th epitope was continued to be synthesized using the Fmoc solid phase synthesis technique until the synthesis was complete.

Synthesis method of G2: Palmitic acid is coupled to the N-terminal peptide of antigen G1 through linker, which forms a lipid tail and produces a self-adjuvant effect. Through this method, the immune effect of the vaccine is enhanced, and the immune effective duration of the immunocastrated vaccine is increased. A new antigen (antigen G2) of GnRH+Th + palmitic acid is synthesized.

### 2.4. The emulsification of the vaccine

I. The oil-in-water vaccine (W/O) was prepared as follows: 8 mg of antigen was weighed using an electronic balance, dissolved in 28 ml of PBS solution, and filtered through a 0.22 μm filter membrane in a sterile environment. The initially filtered 5 mL liquid was discarded, and the other liquid was collected as the aqueous phase. Montanide ISA 61 VG adjuvant was autoclaved and used as the oil phase. 39 oil phases were removed and added to an emulsification cup. The mixture was stirred using a high-speed shear machine and slowly added 21 parts for 16 min, with emulsification for 20 s and stopped for 20 s. The final concentration of the vaccine was 50 μg/mL. The emulsification qualified test was performed, which involved ① dropping a small amount of the vaccine into cold water using a straw to observe if oil droplets spread or not. ② 10 mL of G1 vaccine was added to a centrifuge tube and centrifuged at 3,000 r/min for 15 min with no more than 0.5 mL of water accordingly.

II. To prepare the water-in-Oil-in-water vaccine (W/O/W), 8 mg of antigen was dissolved in 36 mL PBS solution. The solution was filtered through a 0.22 μm filter membrane in a sterile environment, and the initially filtered 5 mL liquid was discarded while the other liquid was collected as the aqueous phase. The Montanide ISA 201 VG adjuvant was autoclaved and used as the oil phase. 27 aqueous phases were removed, and then added to an emulsifying cup, where it was stirred with a high-speed shear machine. 33 parts of oil phase were slowly added, and the mixture was emulsified for 20 s and stopped for 20 s for a total of 16 min. The configured vaccine concentration was 50 μg/mL. The emulsion qualified test involved taking a small amount of vaccine drops with a straw and adding them to cold water. Initially, the drops spread into a cloud shape and finally floated on it. Additionally, 10 mL of the G1 or G2 vaccine was added to a centrifuge tube and centrifuged at 3,000 r/min for 15 min, with no more than 0.5 ml of water accordingly.

### 2.5. Group and treatment of the animals

Forty healthy 13-week-old intact male pigs were randomly assigned to four groups of 10 and housed under experimental conditions (No. LLSC-2021005). The groups were as follows: G2-W/O/W (antigen G2 oil-in-water-in-oil adjuvant group), G1-W/O/W (antigen G1 oil-in-water-in-oil adjuvant group), G1-W/O (positive control antigen G1 oil-in-water adjuvant group), and control (no vaccination). Each animal received 100 μg of antigen in a 2 mL volume, with the first immunization administered at 13 weeks of age and subsequent booster immunizations given at 4-week intervals with the same dose as the initial immunization.

### 2.6. Blood sample collection and processing

Blood samples were collected prior to the first immunization and every 4 weeks thereafter until the end of the experiment. Blood was collected via the anterior vena cava and 4 mL of blood was obtained. The blood was then centrifuged at 3,500 r/min for 15 min at room temperature. After centrifugation, the serum was isolated and stored at 4°C overnight and then stored at −20°C for future use.

### 2.7. Collection of back fat

At the end of the experiment, when the boars were 33 weeks old, 100 g of subcutaneous fat from the back of each boar was collected and stored frozen at −20°C for analysis.

### 2.8. Concentration of GnRH antibody titers in the serum

GnRH IgG antibodies in serum samples were determined by indirect ELISA method established by Tianjin Ringpu Bio-technology Co. Ltd. as follow: The prepared antigen in serum samples was added into 96-well plate, 100 mL contains 50 ng CRM197-4GnRH in each well and then kept in 4°C overnight. Each well was washed by PBST for three times, 1 min in each time. The PBST was poured out and the plate was patted to dryness. 200 μL of 2% BSA was added into each well and kept at 37°C for 2 h. After the reaction, BSA was discarded and each well was washed by PBST for three times, 1 min each time. The liquid in each well was discarded and incubated with 100 μL of diluted goat anti-rat IgG (1:1500) in PBS at 37°C for 1 h. After the reaction, each well was washed by PBST for three times and then treated with 50 μL TMB substrate for 10 min in darkness. After terminated by H_2_SO_4_, the OD value of each well was detected by microplate reader at 450 nm.

### 2.9. Test for testosterone

The determination of testosterone concentration in boar serum was carried out using an iodotestosterone radioimmunoassay cartridge following the instructions provided for the experiment.

### 2.10. Androstenone and fecal determination

As per the report, the threshold for boar odor sensory evaluation was set at 0.2 μg/g for skatole and 0.1 μg/g for androstenone.

#### 2.10.1. Androstenone concentration was determined by the HPLC method

To prepare the samples, the preserved adipose tissue was cut into small pieces using scissors and placed in a beaker. The beaker was heated with a microwave oven to make the fat change into a liquid, which was then transferred into a sealed sample bottle and stored at −20°C. For analysis, samples were placed in a 60°C environment. Then, 150 μL of the sample was added into a 1.5 mL centrifuge tube using a pipette. The tube was then vortexed for 30 s at −20°C, followed by centrifugation at 4500 g for 5 min at −20°C. The resulting supernatant was filtered through a 0.22 μm filter membrane and tested using HPLC.

#### 2.10.2. The skatole concentration was determined by HPLC

Preparation of samples: the same as above.

The chromatographic conditions used are as follows: column temperature: 30°C; mobile phase: ethylene fat: water =40:60 (V: V); flow rate: 1.0 mL/min; excitation wavelength: 270 nm; emission wavelength: 350 nm; sample intake: 20 μL.

### 2.11. Statistical analysis

The data were input into SPSS Statistics 21 statistical software for processing, and their significance was analyzed by an analytical test of variance and multiple comparisons. A one-way ANOVA test of multiple comparisons followed by Dunnett’s post-hoc test was used to evaluate statistical significance between groups. The level of statistical significance for our analyses is 0.05.

## 3. Results

### 3.1. Quality detection of GnRH peptides and analogs

**Figure 2:**
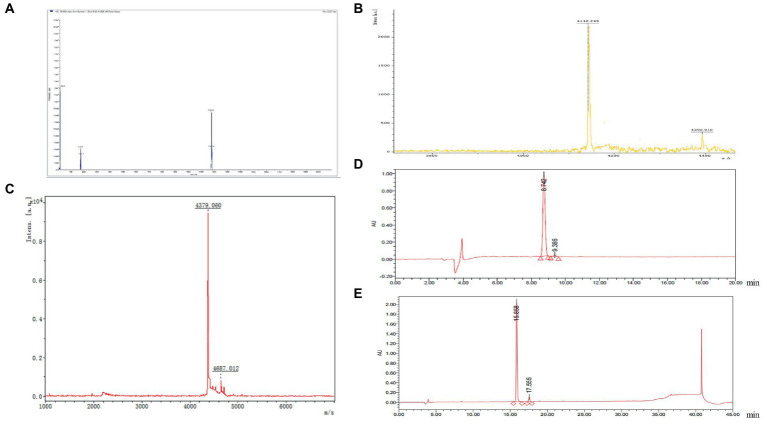
**(A)** The results of mass spectrometry analysis of the molecular weight of GnRH. **(B)** The molecular weight of the G1 polypeptide antigen was determined by mass spectrometry and verified to be 4142.249. **(C)** The molecular weight of G2 peptide antigen was determined by mass spectrometer, and the results showed that it was 4379.06. **(D)** The purity of the purified antigen G1 was analyzed by high performance liquid chromatography, and the purity of the purified antigen G1 reached 98.27%. **(E)** The purity of the purified antigen G2 was analyzed by high performance liquid chromatography, and the purity of the purified antigen G2 reached 95.33%.

To confirm whether the synthesized GnRH polypeptide fragment was the intended target peptide, the fully synthesized peptide was subjected to mass spectrometry to determine its molecular weight and verify if it matched the expected peptide size. The results of the test (as shown in Figure 2A) revealed that the target peptide had the highest peak of the molecular ion fragment with the longest residence time, with a visible peak of 1181.6. This value was consistent with the theoretical molecular weight of 1,181 of the GnRH synthetic peptide that was designed in this experiment. The mass spectrogram displayed only a few miscellaneous peaks, indicating that it met the mass requirements.

The molecular weights of the G1 and G2 peptide antigens were verified using a mass spectrometer to confirm whether they were the target peptides. The synthesized peptides were assessed on the mass spectrometer, and the results showed that the molecular weight of G1 was 4142.249 (Figure 2B), which is nearly consistent with the theoretical molecular weight of the target peptide 4142.78. The molecular weight of G2 was 4379.06 (Figure 2C), which is also nearly consistent with the theoretical molecular weight of the target polypeptide of 4380.13. These results suggest that the G1 and G2 antigens are the desired polypeptides.

The purified antigens G1 and G2 were analyzed for purity using high-performance liquid chromatography. The results showed that the purity of purified antigen G1 was 98.27% (Figure 2D) and the purity of purified antigen G2 was 95.33% (Figure 2E). These values indicate that the purified antigens have high purity, making them suitable for use in the study of the immune castration vaccine.

### 3.2. GnRH immunocastration vaccine stability test

To investigate the stability of the GnRH immunocastration vaccine, four batches of vaccine samples were stored at 4°C for 6, 9, 12, and 15 months. The samples were tested according to the vaccine’s characteristics, and the results shown in Table 1 indicate that the vaccine has stable characteristics.

**Table 1:** The test results before vaccine preservation and after preservation at 4°C for different time.

Group	Keep for long time (Mouth)	Character test		
		Appearance	Dosage form	Precipitated water content (mL)
G1-W/O/W	6	Light yellow transparent liquid	W/O/W	0
	9	Light yellow transparent liquid	W/O/W	0
	12	Light yellow transparent liquid	W/O/W	0.1
	15	Light yellow transparent liquid	W/O/W	0.1
G2-W/O/W	6	Light yellow transparent liquid	W/O/W	0
	9	Light yellow transparent liquid	W/O/W	0
	12	Light yellow transparent liquid	W/O/W	0.1
	15	Light yellow transparent liquid	W/O/W	0.1
G1-W/O	6	Transparent liquid	W/O	0
	9	Transparent liquid	W/O	0.1
	12	Transparent liquid	W/O	0.2
	15	Transparent liquid	W/O	0.2

### 3.3. Safety of the GnRH immunocastration vaccine

In order to conduct a more comprehensive safety assessment of the GnRH immunocastration vaccine, an overdose safety test was performed on minimum-aged target animals (piglets aged 30 to 40 days), with a dose of two times the normal amount. The results of the test, as shown in Table 2, indicate that after administering the overdose vaccination, there were no significant differences observed in body temperature, weight gain, or clinical performance among the test groups during the 14-day observation period following vaccination.

**Table 2:** Safety test results of one-time overdose vaccination for piglets.

Group	No.	Different time after inoculation (d) body temperature (°C)							Initial weight (Kg)	Final weight (Kg)	Average weight gain (Kg)
		1	2	3	4	5	6	7			
G1-W/O/W	1	39.1	38.7	38.6	39.1	39.4	38.8	38.7	9.4	14.1	4.6
	2	39.2	39.4	39.4	38.5	39.6	38.7	39.0	9.1	13.8	
	3	39.3	38.9	38.9	39.1	39.1	39.5	39.1	9.2	13.7	
	4	39.6	39.2	38.8	39.6	38.4	39.6	39.4	9.3	13.9	
	5	38.9	39.4	39.2	39.0	38.9	39.1	39.6	8.9	13.6	
G2-W/O/W	6	39.4	38.9	39.3	39.1	39.4	38.7	38.9	9.2	13.2	4.5
	7	39.3	38.9	38.9	39.2	38.9	39.2	39.3	8.7	13.6	
	8	39.2	39.4	39.0	38.9	39.1	39.1	39.3	9.1	13.7	
	9	38.6	39.3	39.4	39.4	38.7	39.4	39.1	8.7	13.2	
	10	38.7	39.2	39.0	39.6	38.9	39.2	39.5	8.7	13.4	
G1-W/O	11	39.6	39.6	39.6	39.3	39.4	39.5	39.1	9.0	12.9	4.6
	12	39.8	39.2	39.3	39.1	39.1	39.1	39	8.7	13.4	
	13	40.0	39.1	39.2	39.3	39.2	39.5	39.4	8.9	13.6	
	14	39.2	39.5	39.0	39.4	39.1	39.5	39.2	9.1	14.2	
	15	39.0	38.8	38.9	39.1	39.3	39.3	39.0	9.3	13.8	
Control	21	39.3	39.4	39.5	39.3	39.3	39.2	39.5	8.3	13.8	4.6
	22	39.2	39.1	39.2	38.9	39.3	39.3	39.4	9.1	14.0	
	23	39.1	39.3	39.4	39.3	39.5	39.1	39.2	9.4	13.9	
	24	39.9	39.4	39.2	39	39.2	39.1	39.0	9.2	13.3	
	25	40.1	39.8	39.8	39.5	39.3	39.4	39.2	9.5	13.5	

### 3.4. Gnrh antibody titers in the serum

**Figure 3:**
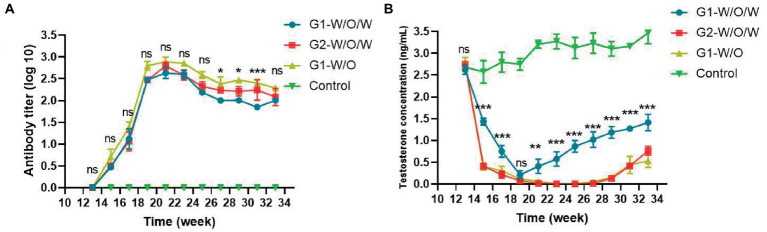
**(A)** Results of GnRH antibody titer in boar serum. The serum antibody titers of boars treated with GnRH were significantly different from the control group (*p* < 0.05). **(B)** After immunization with GnRH, the concentration of testosterone in boar serum was measured (in ng/mL). The results showed that the serum testosterone level in treated boars was lower than 0.1 ng/ml, and there was a significant difference when compared with the control group. The result in the picture shows the comparison between the G1 W-O-W group and the G2 W-O-W group. ns indicated that there was no significant difference. *p < 0.05, **p < 0.01 and ***p < 0.001.

Following the immunization of boars with the GnRH vaccine (Figure 3A), the antibody titers in the immunization group were found to be significantly different compared to the control group (*p* < 0.05). Among the positive control group, G1-W/O group displayed the highest antibody titer value, and the maintained high level began to decline slowly after reaching the peak at week 21. Secondly, G2-W/O/W demonstrated higher titers than G1-W/O/W beginning from 17 weeks of age, and had antibody levels that peaked at 21 weeks of age. The GnRH antibody titer value between G1-W/O and G2-W/O/W vaccines was not significantly different (*p* > 0.05).

### 3.5. The presence of testosterone concentration in the serum

As shown in Figure 3B, Found that the positive control group, the G1-W/O group, At the 19th to 31st weeks of age, testosterone concentration in boar serum was maintained at very low levels (testosterone <0.1 ng/mL), For 12 weeks, Its testosterone concentration was significantly lower than that of the Control group (*p* < 0.05); G1-W/O/W group at 19 to 21 weeks of age, With the Control (p < 0.05), Can last for 2 weeks; The G2-W/O/W group had lower serum testosterone values below 0.1 ng/mL from the 19th to 29th weeks of age, Maintain it for 10 weeks, Serum testosterone concentration begins since 31 weeks of age. It shows that G2 castration works better than untreated G1.

### 3.6. GnRH immunocastration vaccine success rate

Based on the experimental results described above, the serum testosterone concentration was found to be less than 0.1 ng/mL, indicating the success of the vaccine. The specific findings are presented in Table 3.

**Table 3:** The validity statistics of at different periods post-vaccination.

Time (week)	13	15	17	19	21	23	25	27	29	31	33
G1-W/O/W	0/10	0/10	0/10	9/10	10/10	10/10	9/10	9/10	9/10	6/10	2/10
G2-W/O/W	0/10	0/10	1/10	10/10	10/10	10/10	10/10	10/10	10/10	9/10	3/10
G1-W/O	0/10	0/10	4/10	10/10	10/10	10/10	10/10	10/10	10/10	10/10	10/10
Control	0/10	0/10	0/10	0/10	0/10	0/10	0/10	0/10	0/10	0/10	0/10

The table presented in the previous paragraph shows that the G1-W/O vaccine immunization response rate of boars was 100% between 19 and 31 weeks, and the duration of effectiveness was up to 12 weeks. The G1-W/O/W vaccine was found to have an immunization efficiency of 100% in boars at the age of 21 to 23 weeks, with an immunization duration of only 2 weeks. The G2-W/O/W vaccine, in which the GnRH polypeptide was modified with palmitic acid and incubated with oil-in-water–water adjuvant, had an immunization efficiency of 100% with an effective duration of 10 weeks, which was similar to that of G1-W/O. As the market period of boars is typically 6–7 months, as long as the effective immunization period exceeds 8 weeks, it would suffice. Therefore, G2-W/O/W can meet the requirements of a commercial immunocastration vaccine for boars.

### 3.7. Detection of subcutaneous fat androstenone concentration

The results from Table 4 demonstrate that at 33 weeks of age, all three GnRH immunocastration vaccines were able to significantly inhibit the secretion of androstenone in the subcutaneous fat of boars, as compared to the Control group (*p* < 0.05). There was no significant difference observed between the GnRH immunocastration vaccine groups (*p* > 0.05). However, it should be noted that one of the samples from the G1-W/O/W group had an androstenone concentration that exceeded the standard.

**Table 4:** Determined results of androstenone concentration in pig serum.

Group	SD	Minimum value	Maximum value	The number of pigs with androstenone >0.1 μg/g
G1-W/O/W	0.049	0.017	0.157	1/10*
G2-W/O/W	0.017	0.011	0.054	0/10*
G1-W/O	0.018	0.013	0.060	0/10*
Control	0.638	0.085	1.537	9/10

*p < 0.05, all compared to the control group.

### 3.8. Detection of skatole concentration in subcutaneous fat

Furthermore, according to Table 5, all three GnRH immunocastration vaccines were able to inhibit the secretion of skatole in the serum of boars, but one sample from the G1-W/O/W group exceeded the standard.

**Table 5:** Determined results of skatole in pig serum.

Group	SD	Minimum value	Maximum value	The number of pigs with skatole >0.2 μg/g
G1-W/O/W	0.053	0.028	0.214	1/10*
G2-W/O/W	0.264	0.032	0.158	0/10*
G1-W/O	0.034	0.049	0.178	0/10*
Control	0.209	0.071	0.605	8/10

*p < 0.05, all compared to the control group.

## 4. Discussion

The modification of GnRH not only addresses the issue of the immune system’s inability to recognize GnRH hormones, but also enhances the immunogenicity of GnRH while taking into account the specific requirements of its application scenarios. For example, in the case of boar immune castration, it is necessary to maintain a certain duration of immunity to allow the regulation system to perform immune castration effectively (13).

Ladd (14) developed a GnRH immune vaccine by combining the first, sixth, and tenth amino acids of GnRH with tetanus toxin. After immunizing dogs, rabbits, and rats, the antibody titer in serum was determined, and it was found that the best immunogenic effect was achieved by coupling the first amino acid of GnRH with tetanus toxin. Oonk (15) replaced the sixth amino acid of GnRH with Lys, a type D amino acid, and connected the modified GnRH to form a dimer, which was then linked to the carrier protein OVA. Immunization with the resulting vaccine in boars resulted in 100% immunological efficiency. Improvest (Zoetis, United States) is the only commercial product in the world used for boar immunization castration. It removes the first amino acid on the basis of GnRH, yet it still has a certain immune activity. In addition to replacing amino acids, increasing the molecular weight of the antigen can also enhance the ability to stimulate the immune response in the form of polysomy (16). *T* cell epitope peptides are generally below 30 amino acids, have a simple structure, and can be obtained by chemical synthesis (17).

GnRH peptides and their analogs generally have small molecular weights and relatively simple structures, most of which are straight-chain structures resulting in weak immunogenicity and a challenge in stimulating a strong immune response in animals. Additionally, when these polypeptides are directly injected as vaccines, it is difficult to maintain their effects for an extended period of time. However, the use of immune adjuvants and palmitic acid modification can effectively prolong vaccine protection time (18, 19). One of the most mature vaccine adjuvants is the oil emulsion adjuvant (20). This adjuvant, when added to the vaccine and injected into the animal body, can aggregate and store in the injection department, allowing for the slow release of the injected antigen to stimulate the animal body to produce an immune response (21, 22). By adding the GnRH antigen to oil droplets, it is protected from being degraded by proteases in the body, which ensures its immune response stimulation and the extension of the effective duration of immunity after immunization.

It is important for the effective rate of the boar vaccine to reach 100% to ensure that a certain proportion of substandard pigs does not affect the quality of the whole batch of slaughtered pork. To investigate the effectiveness of the G2 antigen immunization and its impact on the immune castration of boars, an experimental model was established using boars as the target animals. The study assessed changes in the boars’ body weight, GnRH antibody titer, testosterone concentration, and levels of androstenone and skatole after immunization. The study found that while the G1-W/O vaccine had an excellent immune effect duration, which resulted in a decline in meat quality. As a result, the less stimulating W/O/W immune adjuvant was selected, but the immune shelf life of the G1-W/O/W vaccine was short. To address this issue, the G1 antigen was modified with a “lipid tail” to prepare the new G2 antigen of “GnRH + Th + palmitic acid.” The G2 antigen was found to be effective in meeting the castration time in the production process while avoiding the stress response. Additionally, the purity of G2 reached 95.33%, providing a guarantee for the development of an immune castration vaccine.

## 5. Conclusion

This study demonstrated that the use of G2-W/O/W castration was effective in achieving the purpose of surgical castration and significantly reducing the concentration of subcutaneous androstenone and skatole in as little as 8 weeks. Moreover, it did not significantly affect the growth rate of pigs and avoided the strong stress response caused by oil emulsion adjuvants, indicating that G2-W/O/W is a safe and effective alternative to surgical castration in swine production.

## Data availability statement

The raw data supporting the conclusions of this article will be made available by the authors, without undue reservation.

## Ethics statement

The animal study was reviewed and approved by Tianjin University of Science & Technology No. LLSC-2021005.

## Author contributions

MW: conceptualization, funding acquisition, project administration, and supervision. HG: data curation, writing—original draft preparation, and visualization. KL: data curation. XF: writing—original draft preparation and methodology. YG: methodology and editing. MB: methodology. YW: data curation, visualization, and data validation. LZ and YS: reviewing and editing. All authors contributed to the article and approved the submitted version.

## Funding

This study was supported by the Construction Project of Tianjin Comprehensive Technology Innovation Center (No. 14ZXLJNC00120) and Key projects supported by Science and Technology in Tianjin (No. 20YFZCSN00120).

## Conflict of interest

HG, LZ, XF, and YG were employed by Tianjin RingpuBio-technology Co., Ltd.

The remaining authors declare that the research was conducted in the absence of any commercial or financial relationships that could be construed as a potential conflict of interest.

## Publisher’s note

All claims expressed in this article are solely those of the authors and do not necessarily represent those of their affiliated organizations, or those of the publisher, the editors and the reviewers. Any product that may be evaluated in this article, or claim that may be made by its manufacturer, is not guaranteed or endorsed by the publisher.
